# Cystatin C predicts the risk of incident cerebrovascular disease in the elderly: A meta-analysis on survival date studies

**DOI:** 10.1097/MD.0000000000026617

**Published:** 2021-07-16

**Authors:** Xin Zheng, Hong-da She, Qiao-xin Zhang, Tong Si, Ku-sheng Wu, Ying-xiu Xiao

**Affiliations:** a Department of Neurology, The First Affiliated Hospital of Shantou University Medical College, Shantou, Guangdong; b Department of Neurology, Xuanwu Hospital, Capital Medical University, Beijing; c Department of Public and preventive medicine, Shantou University Medical College, Shantou, Guangdong, China.

**Keywords:** brain vascular accident, cerebrovascular accident, cerebrovascular apoplexy, cerebrovascular stroke, Cys C, stroke

## Abstract

**Background:**

Stroke is the third leading cause of global year of life lost in all-age and second-ranked cause of disability adjusted life years in middle-aged and elder population. Therefore, it is critical to study the relationship between vascular-related risk factors and cerebrovascular diseases. Several cross-sectional studies have shown that Cystatin C (Cys C) is an independent risk factor for cerebrovascular diseases and levels of Cys C are significantly higher in stroke patients than in healthy individuals. In this meta-analysis, we introduce a Cox proportional hazards model to evaluate the causality between Cys C and the risk of cerebrovascular accident in the elderly.

**Methods:**

We searched PubMed, EMBASE, the Web of Science, and the Cochrane Library from 1985 to 2019 for studies on the relationship between serum Cys C and incidence stroke with Cox proportional hazards models. We conducted a subgroup analysis of the selected studies to determine a connection between atherosclerosis and stroke. Finally, 7 research studies, including 26,768 patients without a history of cerebrovascular, were studied.

**Results:**

After comparing the maximum and minimum Cys C levels, the hazard ratio for all types of stroke, including ischemic and hemorrhagic stroke, was 1.18 (95% confidence interval 1.04–1.31) with moderate heterogeneity (*I*^2^ = 43.0%; *P* = .119) in a fixed-effect model after pooled adjustment for other potential risk factors. In the subgroup analysis, the hazard ratio and 95% confidence interval for Cys C stratified by atherosclerosis was 1.85 (0.97–2.72). As shown in Egger linear regression test, there was no distinct publication bias (*P* = .153).

**Conclusion:**

Increased serum Cys C is significantly associated with future stroke events in the elderly, especially in patients with carotid atherosclerosis. Thus, serum levels of Cys C could serve as a predicted biomarker for stroke attack.

## 1 Introduction

Stroke is the third leading cause of global year of life lost in all-age^[1]^ and second-ranked cause of disability adjusted life years in middle-aged and elder population.^[2]^ In 2017, Stroke leads to 6.1673 million deaths in all-age around the world and ischemic stroke and intracerebral hemorrhage contribute to 2.7474 and 2.9749 million deaths respectively. Although studies based on stroke risk factors and treatments have been performed over the past 25 years, the incidence rate of stroke has increased gradually, especially in some developing countries.^[3]^ Therefore, studying the relationship between vascular-related risk factors and cerebrovascular diseases, or of early intervention of risk factors seems critical.

Chronic kidney disease (CKD) is a risk factor for cerebrovascular accidents and is also closely connected to subclinical cerebrovascular abnormalities (such as asymptomatic cerebral infarction) and cognitive impairment.^[4–6]^ A prospective cohort study in 2009 showed that elevated biomarkers, including serum creatinine (Scr) and Cystatin C (Cys C), were independent predictors of mortality and poor prognosis in cerebrovascular accident in patients with CKD.^[7]^ In another study, renal disorder was also confirmed to be closely connected to the occurrence of stroke. Proper intervention for patients with CKD may prevent stroke events.^[8]^ Brain and kidney diseases may share some similar risk factors and mechanisms in vascular injury^[9]^ since the structure, anatomy, and function of the brain and kidney are similar.^[10]^

In the past, we used Scr to measure renal function. However, Scr is insensitive as it is easily influenced by renal tubular secretion, age, gender, muscle weight, physical activity, and diet.^[11]^ Thus, Scr is less reliable for patients with early stage renal impairment.^[12]^ Cys C is a nonglycosylated basic protein produced by nucleated cells at a constant rate, and it can be freely filtered from the glomerulus. Cys C is completely catabolized by the proximal tubular cells and is not returned to the circulatory system.^[13]^ The application of Cys C to measure the glomerular filtration rate (GFR) has the following advantages: First, Cys C is not secreted in the renal tubules and thus the concentration of plasma Cys C is almost entirely dependent on the estimated GFR; Second, Cys C is not affected by factors such as inflammation, liver diseases, diet, and individual physique (muscle weight) and thus the measured values are more objective.^[12]^ Together, compared with other traditional GFR markers, such as Scr levels and creatinine clearance, Cys C is more sensitive in assessing GFR, especially in detecting mildly reduced GFR.^[12]^

Cys C effectively inhibits cathepsin via regulation of a proteolytic enzyme in vitro and in vivo.^[14]^ Cys C can interact with cathepsin and consequently maintain a dynamic balance between the accumulation and degradation of extracellular matrix proteins under the arterial intima.^[15,16]^ An imbalance in expression between cathepsins (cathepsins S, K, L, or C) and the inhibitor, Cys C, would result in a high incidence of cardiovascular disease, such as atherosclerosis, aneurysm formation, restenosis, and neovascularisation.^[17]^ Multiple cross-sectional case–controlled studies have shown that Cys C is an independent risk factor for increased cardiovascular and cerebrovascular disease in the elderly with normal GFR.^[18,19]^ Cys C levels are significantly higher in stroke patients than in healthy individuals.^[20]^ The aforementioned studies are all cross-sectional studies; thus, the causality between Cys C and stroke may not be well explained. A prospective cohort study can better study the cause as it can directly calculate the risk of a disease with a small selection bias. Here, we introduce a Cox proportional hazards model to evaluate the causality between Cys C and the risk of cerebrovascular accident. This systematic review has been registered on PROSPERO register with a registration number CRD42017067679 (Supplemental Digital Content 1).

## 2 Materials and methods

### 2.1 Identification of studies

We search English-based electronic databases, including PubMed, EMBASE, Web of Science, and the Cochrane Library, for related trials published between January 1, 1985 and July 1, 2019. MeSH terms “Cys C” AND “Stroke” were combined. In-depth searching terms are provided in the supplemental material (Supplemental Digital Content 2). The potential reference lists curated were manually searched to collect all relevant articles. We obtained ethical approval from the ethics committee of the First Affiliated Hospital of Shantou University Medical College.

### 2.2 Select the study

Two reviewers (XZ and YX) independently excluded irrelevant articles based on the titles and abstracts. Full texts were then reviewed to rule out studies that did not meet the 5 inclusion criteria: First, population: participants in the general population without cardiovascular disease (history of stroke, myocardial infarction, heart failure) are included in this study. The categories of stroke included in this study were acute ischemic stroke and acute hemorrhagic stroke. Patients with carotid atherosclerosis were also included for the subtype study. According to data from the Atherosclerosis Risk in Communities study (1987–2001),^[21]^ incidence of stroke in the elderly population were over the age of 45 years. Thus, the selected articles excluded the study which recruited participants below 45 years old, such as the study of young stroke. Second, intervention: observation and follow-up duration of at least 1 year. Third, Comparison: the highest quintile versus the lowest quintile, reported quantitative estimates of the multivariate-adjusted hazard ratio (HR) and 95% confidence interval (CI) for outcomes related to baseline Cys C levels. Fourth, outcomes: overall survival of stroke was evaluated, with the disease confirmed by the medical Centre. Fifth, Cys C levels were tested using the BNII nephelometer.^[22]^ We excluded randomized controlled trial studies, cross-sectional and retrospective case-control studies, reviews, cases report, editorial comments, communications, and reports without sufficient data. Cardiovascular events were confirmed to be related to stroke^[23]^; thus, they would not be excluded from this study.

### 2.3 Data extraction

Two reviewers (XZ and YX) separately collected data from the relevant studies. Differences in the assessment of study eligibility were resolved by group discussion. Data were extracted into a prespecified form and entered into Microsoft Excel. The details of each study included: author, publication date, study country, study design, follow-up duration, number of cases, Cys C level comparison, mean age of cases, HR, 95% CI, and Newcastle–Ottawa Scale (NOS).

### 2.4 Date quality assessment

Evaluation of the methodological quality was based on the NOS. We tested three relevant suspected biases, namely the subject selection process, groups’ comparability level, and the outcomes confirmation. The total NOS star count ranges between 0 and 9. Those that reached a star rate ≥5 were regarded as high-quality outcomes, and <5 stars were regarded as low-quality outcomes.

### 2.5 Statistical analysis

Seven studies were included in the statistical analysis, 6 of which involved all types of stroke events (including ischemic and hemorrhage strokes),^[24–29]^ and 2 studies reported Cys C levels combined with atherosclerosis.^[28,30]^ Cox proportional hazard regression was used to assess the HR and 95% CI for stroke. The maximum concentration of Cys C was compared with the minimum concentration to establish a complete risk assessment model. With the help of Cochran *Q* and *I*^2^ tests, samples with a *P* > .10 after Q tests as well as samples with computed values under 50% were viewed as having no significant heterogeneity among studies; thus, the fixed-effect model was adopted. Otherwise, the random-effect model was adopted.^[31]^ To determine the impact of individual studies on the results obtained, we performed a sensitivity analysis with multi-removal among studies.^[32]^ We used Egger test to assess publication bias (*P* < .05). Then, we used STATA and Review manager to perform this statistical analysis.

## 3 Results

### 3.1 Study search

**Figure 1:**
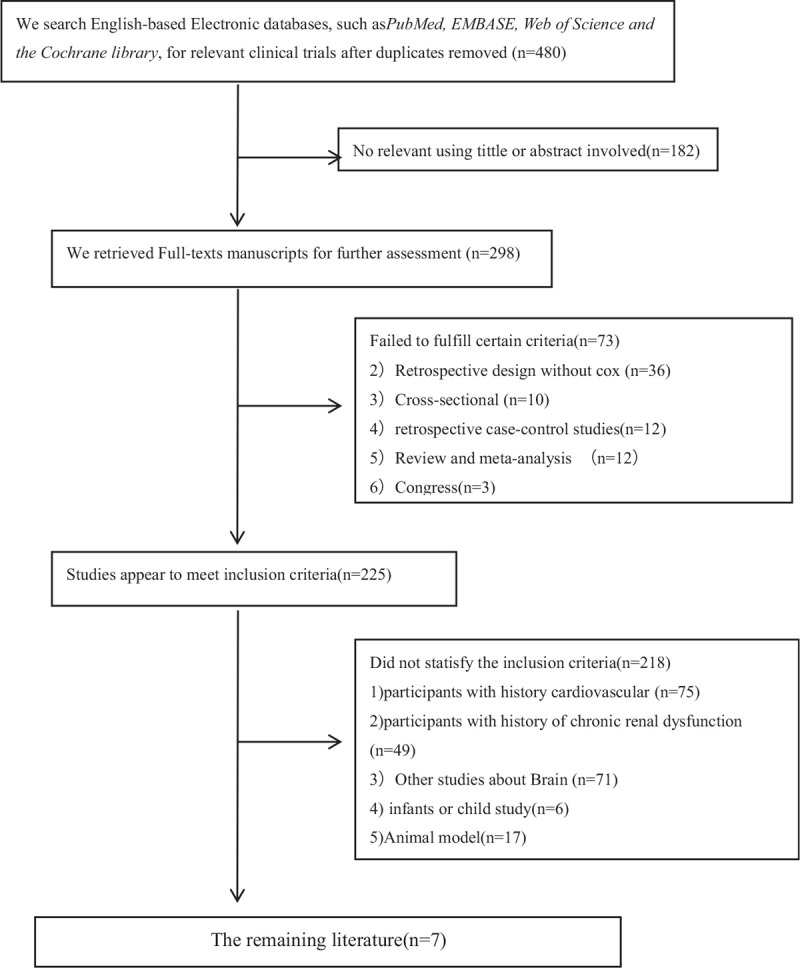
Flow chart of study article selection.

After deleting duplicated studies, a total of 480 articles were obtained. We filtered the articles according to the flow chart in Figure 1. In the first stage, we ruled out 182 articles with irrelevant titles and abstracts. Subsequently, the remaining 298 articles were scanned and screened completely. We excluded 73 articles that did not meet the qualification of literature type, and 218 additional articles did not meet the inclusion criteria or have the exclusion criteria. A total of 7 articles remained in the final stage.

### 3.2 Study characteristics

Seven studies with 26,768 participants were incorporated into this meta-analysis (Table 1). In these 7 studies, 6 articles reported all stroke events and 2 articles reported ischemic stroke. In addition to that, 2 articles reported on the risk of stroke in patients with Cys C combined with carotid atherosclerosis.^[28,30]^ The time of follow-up ranged from 2 to 14.9 years and the sample sizes were from 1004 to 8127 patients.

**Table 1:** Study characteristics included in meta-analysis.

Author	Country	Study design	Follow-up, y	No. of case	Age	Stroke type	Comparison of cys c (highest vs lowest quintile)	HR (95% CI)	Year of publication	Outcome	NOS
Micheal G^[24]^	US	Prospective study	7.4	4637	78	ALL	>1.29 mg/L vs <0.99 mg/L	1.47 (1.09–1.96)	2005	OS	9
Shlipak et al^[25]^	US	Prospective study	9.3	4663	65	ALL	>1.0 mg/L vs <1.0 mg/L	1.21 (1.02–1. 55)	2006	OS	9
Deo et al^[26]^	US	Prospective study	6	3044	70-79	ALL	>1.19 mg/L vs <0.84 mg/L	1.06 (0.89–1. 25)	2008	OS	9
Aguilar et al^[27]^	US	Prospective study	2	3287	78	ALL	>1.28 mg/L *vs* <1. 0 mg/L	1.22 (0. 85–1.74)	2010	OS	9
Hoke et al^[30]^	Australia	Prospective study	3	1004	69	ALL; Atherosolesis	> 0.96 mg/L vs < 0.5mg/L	1.5 (1.07–2.09)	2010	OS	9
Agarwala et al^[28]^	US	Prospective study	14.9	8127	63	ALL; Atherosolesis	—	2.32 (1.53–3.5) adjusted for Cys C ; 2.42 (1.60–3.66) adjusted for Cys C combined with Atherosocesis	2016	OS	9
Garcia-Carretero et al^[29]^	Spain	Prospective study	4.6	2006	52	ALL	—	1.37 (0.68–2.7)	2017	OS	9

CI = confidence interval, HR = hazard ratio, NOS = Newcastle–Ottawa Scale, OR = odds ratio.

### 3.3 Connection between level of Cys C and strokes events

**Figure 2:**
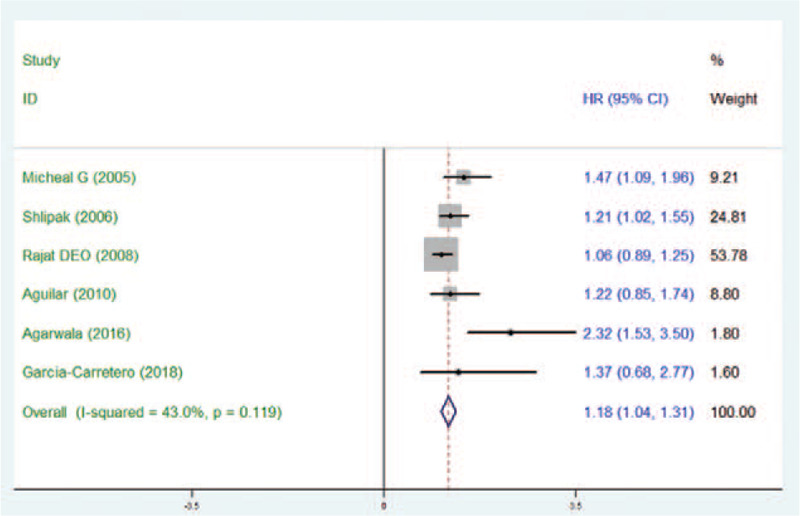
Fixed-effect model comparing the maximum and the minimum levels of Cystatin C.

**Figure 3:**
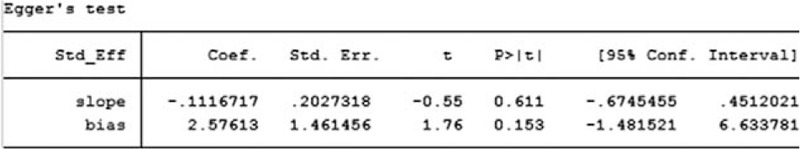
Egger test (*P* = .153).

Six studies reported significant strokes events with a total of 15,746 participants studied (Fig. 2). The pooled adjusted HR comparing the maximum to the minimum Cys C levels was 1.18 (95% CI 1.04–1.31; *P* = .119). We used the fixed-effect(s) model under moderate heterogeneity (*I*^2^ = 43%; *P* = .119). We found no distinct publication bias after testing by Egger's linear regression (*P* = .153) (Fig. 3).

### 3.4 The relationship between Cys C combined with atherosclerosis and stroke

**Figure 4:**
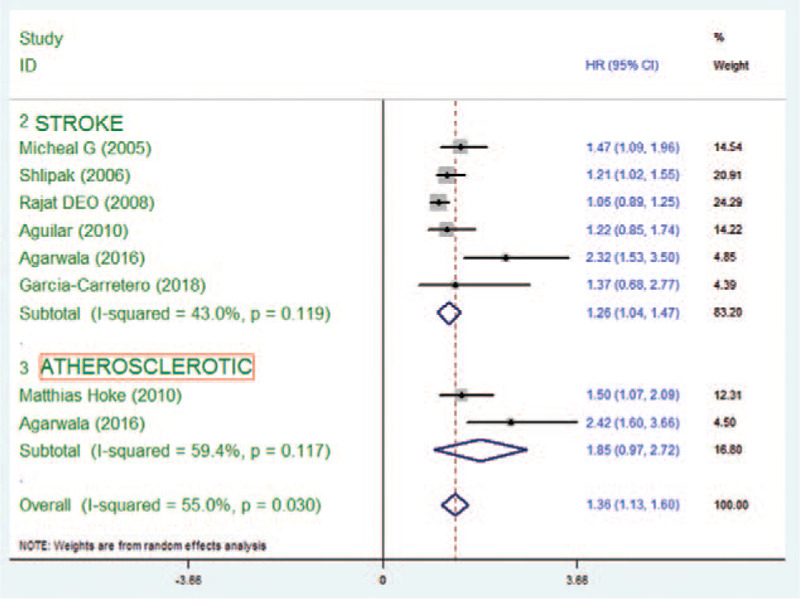
Hazard ratio and 95% confidence intervals for participants with atherosclerosis.

The risk of stroke in patients with Cys C and atherosclerosis was 1.85 (95% CI 0.97–2.72) in the random-effect analysis, with no distinct heterogeneity (*P* = .117) (Fig. 4).

### 3.5 Sensitivity analysis

**Figure 5:**
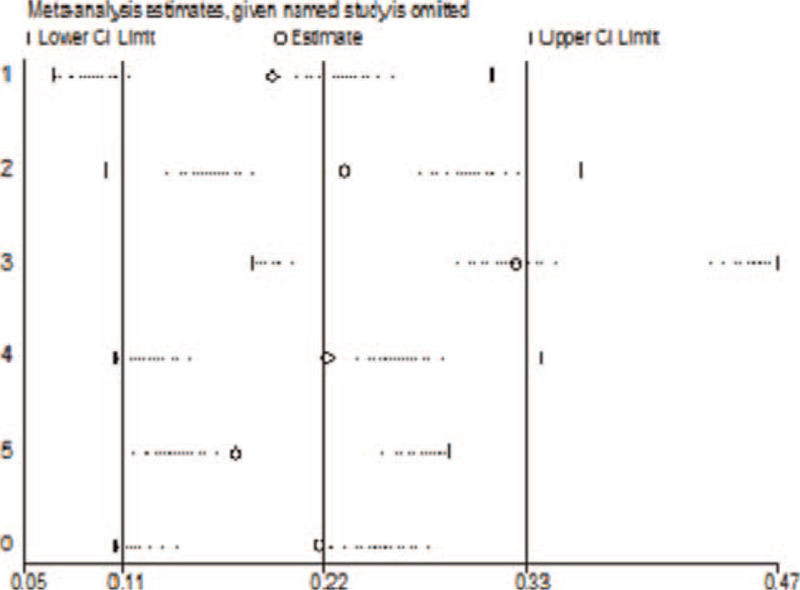
Sensitivity analysis.

A sensitivity analysis was used to evaluate whether the meta-analysis results were stable.^[32]^ The results of the sensitivity analysis of the stroke risk HR showed stability (Fig. 5).

## 4 Discussion

Stroke is the third leading cause of global year of life lost in all-age^[1]^ and second-ranked cause of disability adjusted life years in middle-aged and elder population.^[2]^ Understanding the predictive value of serum biomarkers for cerebrovascular diseases may help with early detection and prevention of cerebrovascular diseases. Numerous studies have shown that Cys C is an independent risk factor for cardiovascular and cerebrovascular diseases.^[18,19]^ However, the causal relationship between Cys C levels and stroke has been well explained since a large number of previous studies have been cross-sectional studies. A prospective cohort study comparing the biomarker Cys C levels with the risk of stroke events has rarely been described.

In this meta-analysis, we aimed to combine multiple relevant studies to assess whether Cys C increases the risk of stroke events. A follow-up study was performed between the exposure group (high-level Cys C) and the non-exposed group (low-level Cys C) to investigate the relationship between the exposure group (high-level Cys C) and the risk of stroke events, and to reveal whether an increased Cys C level causes stroke events. The highest quintiles of Cys C showed a higher risk than the lowest quintiles of Cys C (HR: 1.18, 95% CI: 1.04–1.31; *P* = .119). Based on this basic subgroup analysis, the risk of stroke in participants with Cys C combined with atherosclerosis was 1.85 (95% CI 0.97–2.72). In this study, Cys C is confirmed to be a risk factor for stroke and the elevated levels of Cys C play an important role in predicting stroke events. Increased levels of Cys C may be a harbinger of cerebrovascular disease and may provide novel therapeutic targets for prevention and treatment of stroke. Research about pharmacologically controlled levels of Cys C should be carried out to develop a novel strategy to prevent occurrence of stroke.

In this study, we included study populations with non-cardiovascular disease (history of stroke, history of myocardial infarction, and history of heart failure). The main reasons for using this criterion are as follow: First and foremost, according to statistical methods of cohort studies, the object of the study must have not any of the study outcomes at the beginning. Secondly, as previous studies have shown that Cys C levels are associated with heart disease, the study should recruit people without cardiovascular disease to reduce the impact on the outcome.^[33,34]^ In addition, we excluded those patients with severe medical diseases and peripheral infections which may affect Cys C levels.^[35–41]^ Regarding the time of follow-up, the included literature required observation and follow-up for at least 1 year because the time of follow-up should be selected to meet the need for a <10% loss of visit rate. Also, the 7 final included articles used a consistent laboratory method to measure the Cys C concentration. The reported Cys C concentration in each study was slightly different, but all of original literatures used the highest quartile to the lowest quartile ratio, making the design the same. Meta-analysis of the data is based primarily on their design consistency.

In the included literature, a prospective cohort study carried out by Deo et al enrolled 3075 participants who were followed for >6 years, and 294 cardiovascular events occurred during the follow-up period, including 135 myocardial infarctions and 163 strokes. The authors compared high levels of Cys C (>1.19 mg/L) with low levels of Cys C (<0.84 mg/L). The results again suggest that higher concentrations of Cys C and stroke events are significantly related (HR 1.06; 95% CI 0.89–1.25).^[26]^ In another study, Agarwala et al revealed the relationship between Cys C and atherosclerosis. Patients who had atherosclerosis may have an increased risk of suffering stroke events (HR 2.32, 95% CI 1.53–3.50). Interestingly, the HR and 95% CI for Cys C stratified by atherosclerosis was 2.42 (1.60–3.66), suggesting that the level of atherosclerosis does not significantly influence the association between biomarkers and cerebrovascular disease.^[28]^

Several mechanisms may explain how Cys C level changes are responsible for the increased risk of stroke. First, Cys C plays an important role in the pathogenesis of chronic atherosclerosis.^[33,34,42]^ High levels of Cys C directly affect the balance of extracellular matrix proteins in remodeling the vessel wall,^[43]^ and this becomes a driving force for the occurrence of cerebrovascular disease or cardiovascular disease. Similarly, people with vascular damage-related diseases (such as diabetes,^[44]^ hypertension,^[33,34,42,43]^ and peripheral arterial disease^[45]^) may have elevated Cys C levels,^[34]^ with a potential to suffer from cerebrovascular or cardiovascular disease.^[46]^ Second, the increased level of Cys C, in some cases, is present in renal impairment. Renal dysfunction is also a risk factor for cardiovascular and cerebrovascular events.^[7–10,47]^ Chronic disease treatments play a key role in the prevention of stroke. Third, Cys C may be involved in inflammation,^[35–41]^ which is considered to deteriorate the infarction volume and lead to a poorer prognosis of stroke.^[48]^ Moreover, the membrane protein KCNQ1 reportedly forms a channel complex with KCNE1 or other members of the KCNE family (E1–E5) in the kidney.^[49]^ The KCNQ1 potassium ion channel has been illustrated to be directly involved in cardiovascular diseases.^[50]^ Whether this protein is involved in cerebrovascular disease remains unknown. The lipoprotein lipase has also been identified as a risk factor for cardiovascular disease,^[51]^ suggesting that the related study of apolipophorin may be important.^[52]^

In recent decades, high serum levels of Cys C have not only been linked to cerebrovascular diseases (including stroke and hereditary cerebral hemorrhage with amyloidosis), but also with cognitive impairment (Alzheimer disease, vascular cognitive impairment).^[53]^ Zeng et al^[53]^ recruited a total of 152 patients with acute cerebral infarction to study the connection between high levels of Cys C and vascular cognitive impairment vascular cognitive impairment. The results showed that a high serum level of Cys C might be a risk factor for vascular cognitive impairment after cerebral infarction. Of note, several regulatory mechanisms associated to Cys C-related diseases include atherosclerosis, inflammation,^[54]^ and neurons apoptosis.^[55,56]^ Based on these researches, therapeutic manipulation of Cys C levels or strategically suppressing Cys C to prevent neural disorders should be examined. For instance, Nagai et al^[56]^ found that Cys C might have neurotoxicity characteristics by inducing neuronal cell death, which can be reversed by cathepsin B, a cysteine protease.^[55]^

In contrast, high serum levels of Cys C may have several beneficial effects which should not be underestimated. Neuroprotective effects, for example, were observed in a Parkinson research study.^[57]^ Overexpression of Cys C upregulated the expression of VEGF, NURR1, and the autophagy marker, LC3B, and downregulated expression of the apoptosis marker, CASP3. This promoted angiogenesis in neurodegenerative regions. Expression of Cys C has also been identified to prevent against oxidative stress and neuronal cell death,^[58]^ suggesting that slightly higher the concentrations of Cys C than physiological levels could protect against neuronal cell apoptosis. Interestingly, Pérez-González et al^[59]^ recently found that extracellular vesicles containing high levels of Cys C released from cells has important neuroprotective functions, which paves an avenue for developing a therapeutic tool for central nervous disorders. However, the role of angiogenesis and the reduction of cell apoptosis in cerebral infarction recovery remain elusive.

### 4.1 Limitations and future directions

Most of the participants in the included studies had normal renal function. On this basis, the increased Cys C levels were linearly related to the risk of cardiovascular and cerebrovascular death. Several studies have shown no correlation between race, sex, and risk of cardiovascular events and mortality. In a recent study with 532 patients, Dong et al found a positive correlation between the level of Cys C and age, cardiac diseases (including coronary heart disease, and atrial fibrillation) or lipoproteinemia, while there was no significant correlation with gender, hypertension, diabetes, smoking, and alcohol drinking.^[60]^ Whether there is unrecognized correlation due to insufficient data included remains unknown. Future research can expand the sample size to achieve the correlation analyses among subjects with stroke. In addition, Dong et al's group also classified subtypes of acute cerebral infarction according to TOAST and observed that the majority of stroke subtypes are either large-artery atherosclerosis (LAA) or small vessel occlusion (SAO). Comparing LAA and SAO, LAA had significantly higher Cys C levels than the SAO types, but the difference was not statistically significant. Regrading other subtypes of stroke, such as a cardio embolic stroke and stroke of undetermined etiology, they also had higher levels of Cys C in comparison with the healthy group. Up to date, studies based on younger adults is still rare. Most of the participants in our study are elderly people, and, therefore, the results of this study may not be inferred to young people.

Based on the result from the Grading of Recommendations Assessment (Supplemental Digital Content 3), the analysis conducted in this study indicated that there is moderate heterogeneity, which may be related to age, region, duration of follow-up, or sample size. In addition, Cys C levels are slightly different in each study, so the results may be slightly biased. Meta-analysis is based on original literature to study multiple pieces of date among the literature, so it is impossible to control the confounding factors.

## 5 Conclusion

This meta-analysis used data from 7 previous studies that included patients with elevated levels of Cys C to predict its association with the risk of stroke. The results indicate that increased serum Cys C levels are significantly associated with future stroke events in the elderly, especially in patients with carotid atherosclerosis. The increased levels of Cys C may be a harbinger of cerebrovascular diseases and may be explored for use as a novel therapeutic target for the prevention and treatment of stroke. Based on these findings, therapeutic manipulation of Cys C levels or strategically suppressing Cys C to prevent neural disorders should be explored. In addition, further studies examining the cellular levels should be conducted to reveal the mechanism and the effect of Cys C on angiogenesis and neural cell apoptosis to achieve a satisfactory cerebral infarction treatment. Finally, studies associated with Cys C levels and the risk of stroke events in a younger population needs to be discussed.

## Author contributions

**Conceptualization:** Yingxiu Xiao.

**Data curation:** Hongda She, Tong Si.

**Formal analysis:** Tong Si.

**Investigation:** Qiaoxin Zhang.

**Methodology:** Hongda She, Kusheng Wu.

**Software:** Qiaoxin Zhang, Kusheng Wu.

**Writing – original draft:** Xin Zheng.

**Writing – review & editing:** Xin Zheng, Yingxiu Xiao.

## Supplementary Material

Supplemental Digital Content

## Supplementary Material

Supplemental Digital Content

## Supplementary Material

Supplemental Digital Content
