# Supplementary material for: Cystatin C predicts the risk of incident cerebrovascular disease in the elderly: A meta-analysis on survival date studies
Source: Medicine (Baltimore). 2021 Jul 16;100(28):e26617. doi: 10.1097/MD.0000000000026617 (PMC8284707; doi:10.1097/MD.0000000000026617)
Supplement: Supplemental Digital Content [file medi-100-e26617-s001.doc]

**Supplemental Digital Content 1. Database search strategy**

MeSH terms were combined and classified here: (“Cystatin C”OR “Post-gamma-Globulin”OR “Post gamma Globulin”OR “Neuroendocrine Basic Polypeptide” OR “Basic Polypeptide, Neuroendocrine”OR“Cystatin 3”OR “gamma-Trace” OR “gamma Trace”) AND ("Stroke" OR “Strokes”OR “Cerebrovascular Accident”OR “Cerebrovascular Accidents” OR“CVA (Cerebrovascular Accident) ”OR“CVAs (Cerebrovascular Accident) ”OR“Cerebrovascular Apoplexy ”OR“Apoplexy, Cerebrovascular ”OR “Vascular Accident, Brain ”OR“Brain Vascular Accident ”OR “Brain Vascular Accidents ”OR“Vascular Accidents, Brain ”OR“Cerebrovascular Stroke” OR“Cerebrovascular Strokes” OR “Stroke, Cerebrovascular ”OR “Strokes, Cerebrovascular ”OR“Apoplexy”OR“Cerebral Stroke ”OR“Cerebral Strokes ”OR“Stroke, Cerebral” OR “Strokes, Cerebral ”OR“Stroke, Acute”OR “Acute Stroke ”OR“Acute Strokes ”OR “Strokes, Acute ”OR“Cerebrovascular Accident, Acute ”OR “Acute Cerebrovascular Accident” OR“ Acute Cerebrovascular Accidents ”OR“Cerebrovascular Accidents, Acute”)
