# Supplementary material for: Cystatin C predicts the risk of incident cerebrovascular disease in the elderly: A meta-analysis on survival date studies
Source: Medicine (Baltimore). 2021 Jul 16;100(28):e26617. doi: 10.1097/MD.0000000000026617 (PMC8284707; doi:10.1097/MD.0000000000026617)
Supplement: Supplemental Digital Content [file medi-100-e26617-s002.doc]

**Supporting Information:** Supplemental digital content. Database search strategy: MeSH terms were combined and classified here: (``Cys C''OR ``Post-gamma-Globulin''OR ``Post gamma Globulin''OR ``Neuroendocrine Basic Polypeptide'' OR ``Basic Polypeptide, Neuroendocrine''OR``Cystatin 3''OR ``gamma-Trace'' OR ``gamma Trace'') AND (``Stroke'' OR ``Strokes''OR ``Cerebrovascular Accident''OR ``Cerebrovascular Accidents'' OR``CVA (Cerebrovascular Accident) ''OR``CVAs (Cerebrovascular Accident) ''OR``Cerebrovascular Apoplexy ''OR``Apoplexy, Cerebrovascular ''OR ``Vascular Accident, Brain ''OR``Brain Vascular Accident ''OR ``Brain Vascular Accidents ''OR``Vascular Accidents, Brain ''OR``Cerebrovascular Stroke'' OR``Cerebrovascular Strokes'' OR ``Stroke, Cerebrovascular ''OR ``Strokes, Cerebrovascular ''OR``Apoplexy''OR``Cerebral Stroke ''OR``Cerebral Strokes ''OR``Stroke, Cerebral'' OR ``Strokes, Cerebral ''OR``Stroke, Acute''OR ``Acute Stroke ''OR``Acute Strokes ''OR ``Strokes, Acute ''OR``Cerebrovascular Accident, Acute ''OR ``Acute Cerebrovascular Accident'' OR`` Acute Cerebrovascular Accidents ''OR``Cerebrovascular Accidents, Acute'')
